# COVID‐19 (Omicron strain) hospital admissions from a virtual ward – who required further care?

**DOI:** 10.1111/irv.13108

**Published:** 2023-03-26

**Authors:** Ian Mackay, Megan France, Duncan McAuley, Sean Wing, Mary Wheeldon, Susan Britton, Catherine Todd, Alexandra Pitiris, Leah Barrett‐Beck, Elizabeth Rushbrook, Cameron Bennett, Kate McCarthy

**Affiliations:** ^1^ Royal Brisbane and Women's Hospital Herston Queensland Australia; ^2^ School of Clinical Medicine University of Queensland Herston Queensland Australia; ^3^ Metro North Hospital and Health Service Herston Queensland Australia

**Keywords:** COVID‐19, hospital admissions, Omicron, virtual care

## Abstract

**Background:**

The COVID‐19 virtual ward was created to provide care for people at home with COVID‐19. Given this was a new model of care, little was known about the clinical characteristics and outcomes of patients requiring admission to hospital from the virtual ward platform.

The aims were to characterise hospital admission volume, patient epidemiology, clinical characteristics, and outcome from a virtual ward in the setting of an Omicron (BA.1, BA.2) outbreak.

**Methods:**

A retrospective observational study was performed for all virtual ward patients admitted from 1st January 2022 to 25th March 2022 (over 16 years old). Epidemiological, clinical and laboratory data was reviewed on all patients who required hospital admission.

**Results:**

A total of 7021 patients were cared for on the virtual ward over the study period with 473 referred to hospital for assessment. Twenty‐six (0.4%) patients were admitted to hospital during their care on the ward. Twenty‐two (84.6%) admissions were COVID‐19 related. Fifty three percent of the hospitalised patients were fully vaccinated and 11 had received prior therapeutics for COVID‐19. Shortness of breath was the most common reason for escalation to hospital. Chest pain was the second most common reason and the most common diagnosis after investigation was non‐cardiac chest pain.

**Conclusions:**

Few patients required admission from the virtual ward in the setting of the Omicron variant (BA.1, BA.2) as a direct result of COVID‐19 disease and virtual ward care. Shortness of breath and chest pain were the most common symptoms driving further clinical care.

## 1 INTRODUCTION

The Metro North COVID‐19 Virtual Ward was created to provide care for people at home with COVID‐19 in South‐East Queensland, Central West and Norfolk Island. This covers a population approaching 900 000 and 4157 km^2^. Within these catchments there are 22 public hospitals including 1 quaternary, 1 tertiary and 2 secondary hospitals. The virtual ward structure has been previously described by McCarthy et al. (2022) and had a constant evolution in response to changing needs of COVID‐19 management and demand.^1^ Like a traditional hospital ward, the virtual ward had a list of inpatients and was managed by a multidisciplinary team. However, unlike a traditional hospital ward, the virtual ward patients were at home and consultations were provided over the phone or using telehealth.

Illness severity was characterised by the Australian guidelines for the clinical care of people with COVID‐19 into mild, moderate, severe and critical illness.^2^ The Virtual Ward predominantly managed mild cases of COVID‐19. However, occasionally moderate to severe cases would be identified on review and these cases would be referred for in person assessment and management as required.

To provide care the virtual ward needed to be able to recognise and escalate moderate–severe COVID‐19 cases, complications of COVID‐19 and other medical conditions requiring hospital level care and assessment. In a large multinational observational study, fever, cough, and shortness of breath were the most common symptoms in hospitalised patients for COVID‐19.^3^ Atypical symptoms included nausea, vomiting and abdominal pain for people less than 60 and confusion was the most common atypical symptom for those over 60.^3^ A study by O'Malley, Hansjee^4^ showed that a virtual ward model was useful in following up high‐risk patients discharged from a respiratory ward in a UK hospital. Previous studies have shown that a COVID‐19 virtual ward model of care can relieve pressure on hospitals whilst providing safe management of patients at home.^4^, ^5^, ^6^

The aim of this study was to characterise hospital admission volume epidemiology, clinical characteristics and outcome form a virtual ward model of care as a part of service learning in an Omicron BA.1 and BA.2 outbreak. This knowledge will assist future bed capacity planning and optimise patient care in the virtual ward setting.

## 2 METHOD

### 2.1 Study design

A retrospective assessment was performed for all patients admitted to hospital during their virtual ward admission from 1st January 2022 to 25th March 2022. An admission meant that a patient was admitted to a hospital bed under the care of a specialist. Short stay admissions in the emergency department were excluded. The most predominant Omicron strain during this period was BA.1.

Data was obtained from the Virtual Care Stream (Clinical notes system), the Virtual Ward dashboard (Power BI), The Viewer and iEMR. These are online clinical notes systems where a health professional can visualise patient's previous encounters with public hospitals, including notes and summaries of their previous care. Patients were admitted to the ward as opt‐in model based on a positive chain reaction (PCR) test or positive rapid antigen (RAT) for SARS‐CoV‐2. Outside the opt in model patients were referred by another practitioner (e.g. the emergency department or the General Practitioner) or self‐referred through electronic or phone call platforms. This study was approved by the Royal Brisbane and Women's Hospital Human Research Ethics Committee (Approval number: EX/2022/QRBW/84877).

### 2.2 Virtual Ward and Escalation Process

Patients were admitted to the COVID‐19 Virtual Ward while in isolation at their home. The Virtual ward included administration, nursing, pharmacy, medical and social work staff. An escalation hotline was available to patients after hours.

In this initial consultation patients were risk stratified based on risk of possible disease progression. All patients received daily phone calls and symptoms were assessed with standardised escalation criteria. Higher risk patients received a pulse oximeter. Additional questions were asked for pregnant patients that screened for any issues with pregnancy. The above system allowed escalation of patients to a Medical Officer for review and then to the emergency department if required. Transport to the nearest emergency department was arranged via an ambulance and the Senior Medical Officer of that department was made aware of the patients expected arrival.

Antiemetics, analgesia, antibiotics and oral antiviral therapies when they became available could be delivered to the patient's home and were prescribed according to the Australian National Guidelines.^2^ Sotrovimab and then EVUSHED (tixagevimab and cilgavimab) at a later point were normally given in the outpatient setting but for some time required hospital presentation until this facility was set up. Key changes in ward strategy are listed in Table 1.

**TABLE 1:** Key changes in the virtual ward.

Month	Key changes implemented
January	Sotrovimab access – very limited access (8/1/2022)– increased over time Virtual Dashboard operational (20/1/2022)
February	First budesonide prescription (1/2/2022) Home pulse oximetry introduced (6/2/2022) First Paxlovid (nirmatrelvir and ritonavir) prescription (11/2/2022) Transfer to opt in model of care (14/2/2022) First Lagevrio (molnupiravir) prescription (20/2/2022)
March	14 day follow up for higher risk (immunocompromised) patients (24/3/2022) Very low/low risk category patients discharged from the ward on first consultation (24/3/2022)

Patients were discharged from the virtual ward after 7 days if they met the national guideline criteria relating to symptom improvement.^7^ The Virtual Ward implemented a 14 day total follow up, if they had ongoing symptoms, in the heavily immunosuppressed patient cohort such as lung and liver transplant patients.

### 2.3 Study population

All patients 16 years or over who were admitted to the virtual ward from 1st January 2022 to 25th March 2022 were reviewed. Patients were deemed COVID‐19 positive if they had a self‐reported or confirmed positive PCR test or RAT. Patients were excluded from the study as a hospital admission if they were admitted to hospital to receive an intravenous COVID‐19 therapeutic only or if they were admitted to a short stay ward associated with any department of that hospital.

### 2.4 Data Collection

Data was collected on total numbers of patients admitted to the virtual ward, number of consultations, number of patients who attended an emergency department and those admitted to hospital. The patients admitted to hospital were studied in detail including patient demographics, vaccination status, comorbidities (including immunocompromise), COVID‐19 testing results, reason for escalation, hospital assessment, pathology results, hospital admission treatments, Virtual Ward disposition and treatment outcome. Vaccination status was defined per the Australian Technical Advisory Group on Immunisation (ATAGI).^8^ Day 0 of COVID‐19 was defined as the date the patient was diagnosed or day of first symptoms (not more than 48 hours prior to diagnosis date).^7^ Immunocompromise was defined per ATAGI and included medical conditions such as active haematological malignancy, immunosuppressive therapy, organ transplant with immunosuppressive therapy and primary immunodeficiency syndromes.^9^ Mortality data was obtained (in‐hospital or within 30 days of discharge) and readmission was defined as a further admission to a hospital (not a short stay unit) within 30 days.

### 2.5 Data Analysis

The data was analysed using SPSS Statistics 27. Descriptive statistics were expressed as a number (%) and mean or median for continuous variables.

## 3 RESULTS

### 3.1 Virtual ward admissions, emergency department presentations, and hospital admission rate

A total of 7021 people were actively managed by the virtual ward over the study period. A total of 473 of those patients, 6.7%, attended an emergency department for assessment as a result of escalation of their care by the virtual ward or self‐escalation. Of those, 26 patients (0.4% of the total ward patients), were admitted to a hospital for further care (Table 2).

**TABLE 2:** Numbers of patients admitted to the virtual ward, attendance to an emergency department due to escalation, and admitted to a hospital from January to March 2022.

	January	February	March
**Virtual ward admissions, n (n = 7021)**	3894	1399	1728
**Escalations, n (% of total admissions that month) (n = 473)**	182 (4.7%)	124 (8.9%)	167 (9.7%)
**Hospital admissions, n (% of total admissions that month) (n = 26)**	6 (0.2%)	13 (0.9%)	7 (0.4%)

A total of 26 patients were admitted to hospital during the period of the study. Patient characteristics are summarised in Table 3. Note this included hospital admissions for all causes, not only COVID‐19 admissions.

**TABLE 3:** Baseline characteristics of Hospitalised patients (n = 26).

Baseline characteristics	All hospitalised patients
**Median age, years (range)**	62 (18–89)
**Female sex, n (%)**	17 (65.4)
**Vaccination status**	
Fully vaccinated and up to date, n (%)	11 (42.3)
Fully vaccinated, n (%)	3 (11.5)
Partially vaccinated, n (%)	8 (30.8)
Unvaccinated, n (%)	4 (15.4)
**Pregnant, n (%)**	2 (7.6)
**Risk category**	
Very High, n (%)	13 (50.0)
High, n (%)	11 (42.3)
Moderate, n (%)	2 (7.7)
Low/Very Low, n (%)	0 (0)
**Immunocompromise, n (%)**	5 (19.2)
**Median Charlson Comorbidity Index, index (range)**	2 (0–8)
**Other at‐risk comorbidities**	
Hypertension, n (%)	12 (46.2)
Diabetes, n (%)	6 (23.1)
Obesity, n (%)	4 (15.4)
Chronic Obstructive Pulmonary Disease, n (%)	3 (11.5)
Asthma (Moderate to Severe), n (%)	4 (15.4)
**Virtual ward treatments**	
Budesonide, n (%)	7 (26.9)
Increased usual inhaled corticosteroid dose, n (%)	2 (7.7)
Sotrovimab, n (%)	1 (3.8)
Molnupiravir, n (%)	1 (3.8)
Paxlovid, n (%)	0 (0.0)
Nil, n (%)	15 (57.7)

Table 4 shows the escalation characteristics of the patients who went on to be admitted to hospital. Five (19.2%) patients escalated themselves to hospital for care. Shortness of breath was the most common reason for escalation. The second most common reason was chest pain, the diagnoses of which are listed in Table 5.

**TABLE 4:** Escalation characteristics (in patients admitted to hospital) (n = 26).

Escalation characteristics	
**Median COMPASS score (on day of escalation) (n = 21)**	6
**Self‐escalated, n (%)**	5 (19.2)
**Median day of COVID‐19 illness when escalated, day (range)** *	5.5 (2–16)
**Escalation symptoms**	
Presence of chest pain, n (%)	10 (38.5)
Presence of shortness of breath, n (%)	13 (50)
Other symptoms	9 (34.6)

* Included day 0.

**TABLE 5:** Chest pain diagnoses of patients admitted to hospital (n = 10).

Chest pain diagnoses	Number of patients (%)
Community acquired pneumonia	2
Lower respiratory tract infection	2
Asthma exacerbation	2
Non‐cardiac chest pain, spontaneously resolved	3
Atelectasis	1

Other reasons for escalation included per vaginal bleeding (n = 1), flank pain (n = 1), dysuria (n = 1), desaturation on pulse oximeter (n = 2), fevers/severe fatigue (n = 1), reduced oral intake (n = 2) and tingling of the hands and feet (n = 1).

### 3.2 Admission Characteristics and Outcomes

The summary of the admission data is shown in Table 6.

**TABLE 6:** Admission summary (n = 26).

Admission characteristics	
**Median length of Hospital stay, days (range)**	3 (0–21)
**ICU admissions, n (%)**	1 (3.8)
**COVID‐19 Illness Severity**	
Mild, n (%)	6 (23.1)
Moderate, n (%)	10 (38.5)
Severe, n (%)	7 (26.9)
Critical, n (%)	2 (7.7)
**COVID‐19 related admissions, n (%)**	22 (84.6)
**COVID‐19 Treatments during hospitalisation**	
Budesonide/Other Inhaled Corticosteroid, n (%)	5 (19.2)
Dexamethasone, n (%)	11 (42.3)
Oral steroids, n (%)	10 (38.5)
Remdesivir, n (%)	1 (3.8)
Baricitinib, n (%)	2 (7.7)
Oxygen therapy, n (%)	10 (38.5)
**Admission Outcome**	
In‐hospital mortality, n (%)	1 (3.8)
Discharge to home, n (%)	25 (96.2)
**30‐day outcome**	
Readmission within 30 days, n (%)	1 (4)
30‐day mortality, n (%)	0 (0)

There were no instances of cardiac chest pain or pulmonary embolus substantiated by relevant imaging/pathology results. One patient was admitted for an asthma exacerbation and tested negative for COVID‐19, having already completed the 7‐day isolation period and therefore was classed as an unrelated admission. Admissions not directly related to COVID‐19 included antepartum haemorrhage (n = 1), Epstein Barr Virus infection (n = 1) and urinary tract infection (n = 1).

## 4 DISCUSSION

A total of 7021 patients were cared for on the virtual ward over the study period. Only 0.4% of those patients were admitted to hospital. There were 473 (6.7%) patients escalated to the emergency department for further assessment. There was no reduction in the number of patients admitted to hospital per month for the three months that this data was collected indicating that the changes in process of the virtual ward did not change admission/escalation rates.

One reason for the low admission rate is potentially due to the mass vaccination rollout as this has been shown to significantly reduce risk of hospital admission.^10^ Previous studies have shown that there is an increase in hospital mortality with floods of patients indicating the need for streamlined pathways for admission.^11^ Studies have also shown that the COVID‐19 pandemic has decreased hospital presentations, for example in three hospitals in America showing reduced psychiatric presentations and hospitalisations.^12^ There was also a decreased in presentations of Chronic Obstructive Pulmonary Disease exacerbations due to physical and behavioural measures taken to limit COVID‐19 transmission.^13^

Fifty three percent of the patients admitted to hospital from the virtual ward were fully vaccinated. This is significantly lower than the population vaccination rate at this time which was gradually increasing and by 20 March 2022 was greater than 80% of the community population.^14^ Almost 60% of the cohort were not able to receive therapeutics due to their unavailability or less commonly patent refusal. Thus, therapeutics did not pay a major role in prevention of hospital admission.

Shortness of breath was the most common reason for a Medical Officer to escalate a patient for in‐hospital assessment, despite the availability of pulse oximeters. As this was a new unvalidated process at this time clinician discretion was utilised as to whether the readings changed patient care and if a patient was kept at home when experiencing this symptom with normal oximetry.

Chest pain was a common complaint amongst patients admitted to hospital, however there were no sinister causes of the chest pain found on further investigation. The workup for these patients included basic bloods (full blood count, electrolytes, liver function, kidney function), troponin, chest x‐ray and an electrocardiogram. Three of the 10 patients with chest pain had a d‐dimer and one had a CT pulmonary angiogram. The most common diagnosis amongst this cohort was non‐cardiac chest pain that spontaneously resolved but also included lower respiratory tract infection and asthma exacerbation. Knowing this would allow the virtual ward to potentially reduce the number of escalations to hospital in patients with chest pain in the future. This represents greater familiarity of disease manifestations form the Omicron variant.

In‐hospital mortality for this group was 3.8% (n = 1) and ICU admissions was 3.8% (n = 1). This is largely different to the epidemiology and clinical characteristics of COVID‐19 in Wuhan in 2019 with the alpha variant where there were 138 hospitalised patients with COVID‐19 pneumonia, with 26% needing ICU treatment and a mortality of 4.3%.^15^ Previous studies have shown that the delta outbreak in unvaccinated population would lead to a greater burden on the health care system.^16^ This study has shown that the burden on the hospital system was very low with the implementation of the virtual ward.

Study limitations included that, in the latter half of the study, the model of care was an “opt in” one and thus patients admitted to the virtual ward were self‐selected. The change in patients' management that occurs with greater familiarity with disease manifestations of a new variant and greater familiarity with technology as it is introduced such as pulse oximetry. Also, availability of therapeutics increased in the latter half of the study period.

## 5 CONCLUSIONS

From the virtual ward setting hospital presentations to the emergency department and for admission was a small percentage of the cohort. This was in the setting of a vaccination rate of 57%, limited therapeutics for most of the study period and when Omicron (BA.1/BA.2) was the predominant strain. Shortness of breath and chest pain were the most common symptoms resulting in hospital admission.

## AUTHOR CONTRIBUTION STATEMENT

**Ian Mackay:** Conceptualization‐Supporting, Data curation‐Lead, Formal analysis‐Lead, Investigation‐Lead, Methodology‐Supporting, Project administration‐Equal, Resources‐Equal, Supervision‐Supporting, Validation‐Supporting, Visualization‐Lead, Writing – original draft‐Lead, Writing – review & editing‐Supporting. **Megan France:** Conceptualization‐Supporting, Formal analysis‐Supporting, Resources‐Equal, Supervision‐Supporting, Validation‐Supporting, Visualization‐Supporting, Writing – review & editing‐Supporting. **Duncan McAuley:** Methodology‐Supporting, Resources‐Equal, Validation‐Supporting, Writing – review & editing‐Supporting. **Sean Wing:** Methodology‐Supporting, Resources‐Equal, Writing – review & editing‐Supporting. **Mary Wheeldon:** Methodology‐Supporting, Resources‐Equal, Writing – review & editing‐Supporting. **Susan Britton:** Methodology‐Supporting, Resources‐Equal, Writing – review & editing‐Supporting. **Catherine Todd:** Data curation‐Supporting, Writing – review & editing‐Supporting. **Alexandra Pitiris:** Data curation‐Supporting, Writing – review & editing‐Supporting. **Leah Barrett‐Beck:** Resources‐Equal, Writing – review & editing‐Supporting. **Elizabeth Rushbrook:** Resources‐Equal, Writing – review & editing‐Supporting. **Cameron Bennett:** Conceptualization‐Supporting, Formal analysis‐Supporting, Funding acquisition‐Equal, Project administration‐Equal, Resources‐Equal, Supervision‐Supporting, Validation‐Supporting, Visualization‐Supporting, Writing – review & editing‐Supporting. **Kate McCarthy:** Conceptualization‐Lead, Data curation‐Supporting, Formal analysis‐Supporting, Funding acquisition‐Equal, Investigation‐Supporting, Methodology‐Lead, Project administration‐Equal, Resources‐Equal, Supervision‐Lead, Validation‐Lead, Visualization‐Supporting, Writing – original draft‐Supporting, Writing – review & editing‐Lead.

## DECLARATIONS

No funding was received for this study.

Declarations of interest: none.

All relevant ethical guidelines have been followed. Ethics approval was granted by the Royal Brisbane and Women's Hospital Human Research Ethics Committee (Approval number: EX/2022/QRBW/84877).

There was no material reproduced from other sources. This study was not registered on a clinical trials registry.

### PEER REVIEW

The peer review history for this article is available at https://publons.com/publon/10.1111/irv.13108.

## Data Availability

The data that support the findings of this study are available on request from the corresponding author. The data are not publicly available due to privacy or ethical restrictions.
